# Identification of Major and Minor QTL for Ecologically Important Morphological Traits in Three-Spined Sticklebacks (*Gasterosteus aculeatus*)

**DOI:** 10.1534/g3.114.010389

**Published:** 2014-02-13

**Authors:** Jun Liu, Takahito Shikano, Tuomas Leinonen, José Manuel Cano, Meng-Hua Li, Juha Merilä

**Affiliations:** * CAS Key Laboratory of Animal Ecology and Conservation Biology, Institute of Zoology, Chinese Academy of Sciences (CAS), Beijing 100101, China; † Ecological Genetics Research Unit, Department of Biosciences, University of Helsinki, Helsinki, Finland; ‡ Research Unit of Biodiversity (UO-CSIC-PA), Edificio de Investigación, C/Gonzalo Gutiérrez Quirós s/n, 33600, Mieres, Spain

**Keywords:** body shape, *Gasterosteus aculeatus*, genetic linkage map, morphology, QTL

## Abstract

Quantitative trait locus (QTL) mapping studies of Pacific three-spined sticklebacks (*Gasterosteus aculeatus*) have uncovered several genomic regions controlling variability in different morphological traits, but QTL studies of Atlantic sticklebacks are lacking. We mapped QTL for 40 morphological traits, including body size, body shape, and body armor, in a F_2_ full-sib cross between northern European marine and freshwater three-spined sticklebacks. A total of 52 significant QTL were identified at the 5% genome-wide level. One major QTL explaining 74.4% of the total variance in lateral plate number was detected on LG4, whereas several major QTL for centroid size (a proxy for body size), and the lengths of two dorsal spines, pelvic spine, and pelvic girdle were mapped on LG21 with the explained variance ranging from 27.9% to 57.6%. Major QTL for landmark coordinates defining body shape variation also were identified on LG21, with each explaining ≥15% of variance in body shape. Multiple QTL for different traits mapped on LG21 overlapped each other, implying pleiotropy and/or tight linkage. Thus, apart from providing confirmatory data to support conclusions born out of earlier QTL studies of Pacific sticklebacks, this study also describes several novel QTL of both major and smaller effect for ecologically important traits. The finding that many major QTL mapped on LG21 suggests that this linkage group might be a hotspot for genetic determinants of ecologically important morphological traits in three-spined sticklebacks.

Understanding the genetic architecture of quantitative traits can provide important insights toward elucidating the molecular basis of adaptation and evolution (Merilä and Sheldon 1999; Erickson *et al.* 2004; Phillips 2005; Ellegren and Sheldon 2008). Quantitative trait locus (QTL) mapping represents a classical method to uncover genomic regions controlling variation in phenotypic traits, as well as to gain insights into the distribution of QTL effect sizes of ecologically and evolutionarily important traits (Lynch and Walsh 1998; Slate 2005). Although there are potential problems and biases involved in QTL mapping methods (*e.g.*, Beavis 1994; Slate 2005, 2013), they have succeeded in revealing surprisingly fine-grained (*e.g.*, Colosimo *et al.* 2005) and relatively accurate (*e.g.*, Price 2006) information on the location of genetic factors controlling variation in quantitative traits.

Morphological traits, such as those portraying variation in organismal size and shape, typically are complex quantitative traits under the control of an interacting network of genes and environmental factors (Wu and Lin 2006; Mackay *et al.* 2009). The genetic basis of morphological variation has been studied extensively in domestic animals (*e.g.*, Mott *et al.* 2000; Karlsson and Lindblad-Toh 2008) and plants (*e.g.*, Nordborg and Weigel 2008; Bergelson and Roux 2010) but less so in wild vertebrates (reviews in: Slate 2005; Slate *et al.* 2010). One of the reasons for this paucity of studies in wild vertebrates relates to the high expense and difficulty in raising large numbers of individuals in controlled conditions. However, such studies have now become possible in many emerging model organisms, including the three-spined stickleback, *Gasterosteus aculeatus*, which can be bred and raised in controlled conditions with relative ease.

The three-spined stickleback is emerging as a model system to study adaptation and speciation in vertebrates (Bell and Foster 1994; McKinnon and Rundle 2002; McKinnon *et al.* 2004; Gibson 2005; Barrett 2010). This small marine teleost fish has repeatedly and independently colonized numerous freshwater habitats across the northern hemisphere since the last ice age and adapted rapidly to new environments often in a parallel fashion (Bell and Foster 1994; McKinnon and Rundle 2002). The derived freshwater populations exhibit morphological phenotypes distinct from their marine ancestors, including changes in body size and shape, armor, and trophic morphology (Lavin and McPhail 1986; Bell and Foster 1994; Walker and Bell 2000). The genetic bases of these phenotypic traits have been the focus of many QTL-mapping studies in the last decade (Peichel *et al.* 2001; Colosimo *et al.* 2004; Cresko *et al.* 2004; Shapiro *et al.* 2004; Kimmel *et al.* 2005; Albert *et al.* 2008; Kitano *et al.* 2009; Greenwood *et al.* 2011; Rogers *et al.* 2012). These studies have identified several genes and genomic regions contributing to variation in quantitative traits and thereby provided important insights into the genetic mechanisms of morphological divergence and adaptation. However, with the exception of two recent case studies, which focused on the genetic architecture of a set of correlated traits relating to body shape (Albert *et al.* 2008; Rogers *et al.* 2012), most of these studies have focused on the genetic architecture of a single trait or phenotype (*e.g.*, Colosimo *et al.* 2004). In addition, almost all of the previous investigations have used mapping crosses of Pacific origin (*i.e.*, Canada, United States, or Japan; Supporting Information, Table S1), whereas few QTL studies have used populations of Atlantic origin (but see Shapiro *et al.* 2004; Coyle *et al.* 2007). Because QTL can be lineage- and even population-specific (Symonds *et al.* 2005; Chen and Ritland 2013), a comparative approach is necessary to distinguish ancestral adaptive variation (*i.e.*, shared across lineages) from more recently evolved (*i.e.*, lineage specific) adaptations.

The main aim of this study was to conduct a QTL-scan in three-spined sticklebacks originating from the Atlantic lineage, with particular focus on ecologically important morphological traits: body size, body shape, and armor. To this end, we scanned for QTL associated with these traits using 131 microsatellite markers across the whole genome in a F_2_ full-sib cross generated from marine and freshwater populations. We discuss the results in the context of previous research done in Pacific three-spined sticklebacks. In addition, we compared our genetic linkage map with the physical map generated by BLAST searches against the *G. aculeatus* genome. Apart from adding new dimensions to the understanding of the genetic architecture of phenotypic traits in the three-spined stickleback, our findings provide insights into the distribution of QTL effect sizes in a number of ecologically important morphological traits.

## Materials and Methods

### Sampling and rearing

One F_2_ full-sib family consisting of a total of 190 individuals was generated for the QTL mapping. In brief, a female marine stickleback (F_0_) collected from the Baltic Sea (Helsinki; 60°12′N, 25°11′E) was crossed with a male freshwater stickleback (F_0_) from Lake Pulmanki (Lapland; 69°58′N, 27°58′E) in Finland. The female originated from a population that most likely represents the ancestral northern European marine form (Leinonen *et al.* 2011a). The detailed procedures for crossing F_0_ grandparents have been described in Leder *et al.* (2010). The resulting F_1_ offspring were reared to maturity in the aquaculture facilities of the University of Helsinki. The F_1_ progeny were first raised at 17° for 3 mo and then transferred to 4° for 5 mo to simulate overwintering, and then transferred back to 17° to stimulate breeding. One F_1_ female and one F_1_ male were further crossed to obtain F_2_ progeny. The same F_1_ couple was crossed naturally five times, thus the five broods (brood 1: 69 fish; brood 2: 22 fish; brood 3: 22 fish; brood 4: 44 fish; brood 5: 33 fish) amounted to a total of 190 offspring. Each brood was divided into two to six blocks, with an average of 11 fish per block. Each of the 17 blocks was held in a 27-L aquarium. The F_2_ progeny were reared at 17° for 3 mo, fed *ad libitum* with frozen chironomid larvae, and then killed with an overdose of MS-222. All the samples used in the following analyses were preserved in 96% ethanol and stored horizontally to avoid bending. The samples were then fixed and stained with alizarin red solution for phenotyping. Sex of the F_2_ offspring was identified by two sex-specific molecular markers (GAest31 and Stn190) based on Natri *et al.* (2013).

### Morphometric data collection

**Figure 1:**
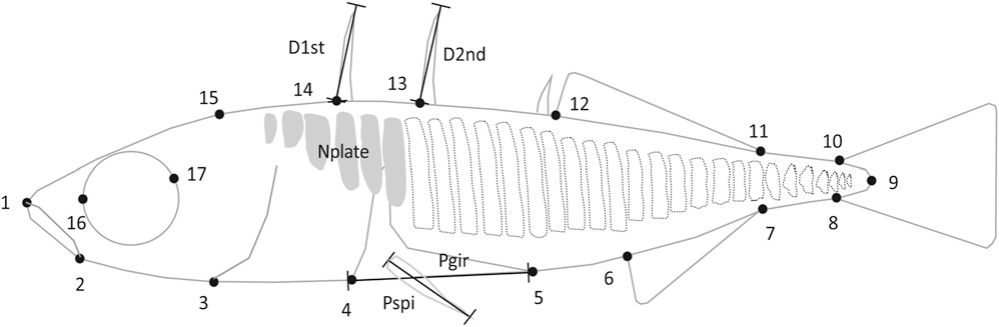
Landmark locations for body shape and metric and meristic traits. Dotted outlines depict the lateral plates present in marine populations but reduced in freshwater populations. The traits and landmarks are described in the main text. Nplate, number of lateral plates; Pgir, pelvic girdle length; Pspi, pelvic spine length; D1st, length of the first dorsal spine; D2nd, length of the second dorsal spine.

Fish preparation, image acquisition, and morphometric measurements followed the procedures described in Leinonen *et al.* (2006). Traditional and landmark-based geometric morphometrics methods were used to quantify variation in body size, body shape, and armor (Figure 1). In brief, a total of 17 landmarks (for the details, see Leinonen *et al.* 2011a) were digitized on the left side of each sample using tpsDig version 2.10 (Rohlf 2006), and the aligned coordinate values (X and Y) were recorded. Because X and Y landmark coordinates usually map to different locations on linkage groups (Albert *et al.* 2008; Rogers *et al.* 2012), they were treated as separate variables. Values of the centroid size (Csize) also were recorded as a measure of body size (Bookstein 1986), and it was strongly correlated with standard length (*r_s_* = 0.99, n = 185, *P* < 0.001). Csize is the square root of the sum of the squared distances from the measured landmarks to their centroid. This measure is independent of any potential random measurement error in the landmarks, being a very robust measure of geometric size (Mitteroecker and Gunz 2009). In addition, the number of lateral plates on both sides (Nplate; the total number of lateral plates on myomeres 1−33) was quantified from photographs, and four metric variables were measured with a digital caliper: (1) length of the first dorsal spine (D1st), (2) length of the second dorsal spine (D2nd), (3) average length of the left and right pelvic spines (Pspi), and (4) pelvic girdle length (Pgir). Therefore, a total of 40 morphometric variables (six metric and meristic traits, including Csize and 34 landmark coordinates) were used in the following QTL mapping analyses.

### DNA extraction and microsatellite genotyping

Genomic DNA was extracted from fin clips using a silica-based purification method (Elphinstone *et al.* 2003) after proteinase K digestion. A total of 131 microsatellite markers (Table S2), previously isolated for three-spined sticklebacks (Largiadèr *et al.* 1999; Peichel *et al.* 2001; Heckel *et al.* 2002; Colosimo *et al.* 2004; Colosimo *et al.* 2005; Miller *et al.* 2007; Mäkinen *et al.* 2008), were genotyped for the grandfather, two F_1_ parents and the 190 F_2_ progeny. Genotype data of the grandmother (F_0_) was inferred from the genotypes of twenty F_1_ offspring due to sample degradation. Note that primers of all the “GAest” markers except GAest46 were from Mäkinen *et al.* (2008). Primers for GAest46 were forward (5′-AGT GAT CAA TAA CCA GAA GGA G-3′) and reverse (5′-CGA TAT GCT TTC ATT GTA TTT G-3′; H. Mäkinen, unpublished data). Polymerase chain reactions (PCRs) were conducted in a 10-μL volume consisting of 1× QIAGEN Multiplex PCR Master Mix (QIAGEN), 0.5× Q-Solution, 2 pmol of each primer, and *ca*. 20 ng of template DNA. The forward primers were labeled with FAM, HEX, or TET fluorescent dye, and a GTTT-tail was added to the 5′-end of the reverse primers to promote adenylation (Brownstein 1996). PCR conditions were as follows: initial denaturation at 95° for 15 min, followed by 30 cycles of 30 sec at 94°, 90 sec at 53° and 60 sec at 72°, and a final extension at 60° for 5 min. PCR products were resolved using a MegaBace 1000 capillary sequencer (Amersham Biosciences) with ET-ROX 550 size standard (Amersham Biosciences) and were analyzed using Fragment Profiler 1.2 (Amersham Biosciences). All the makers were checked for null alleles by using Micro-Checker v.2.2.3 (Van Oosterhout *et al.* 2004).

### Linkage analysis and genetic linkage map construction

A genetic linkage map was constructed using JoinMap v.4.0 software (Van Ooijen 2006). The cross-pollinator population type, which allows for segregation of up to four alleles per locus, was used. Linkage phases of the loci were determined automatically by the software. Test for locus segregation distortion was implemented by the χ^2^ test. All the microsatellites were assigned to linkage groups with a relatively stringent two-point logarithm of odds (LOD) score ≥ 4.0. A map for each linkage group was created using a regression mapping module with the following parameters: a recombination frequency < 0.499, and a LOD score > 3.0. Map distances (centiMorgans, cM) were calculated using the Kosambi mapping function. All the other parameters were set to default values.

### BLAST searches

To map the physical locations of the microsatellites in the genome, BLAST searches were performed against a three-spined stickleback genome assembly of Roesti *et al.* (2013) by using the BLAST module within BioEdit v.7.1.8 (Hall 1999). This genome assembly improved version of the Broad S1 assembly of *G. aculeatus* genome in Ensembl Genome Browser (Roesti *et al.* 2013; http://datadryad.org/resource/doi:10.5061/dryad.846nj). BLAST hits were obtained using the BLASTN searching tool with default settings. The expectation value 1×*e*^-100^ was first used to get a unique hit. When no hit was found for a sequence, the expectation value decreased to 1×*e*^-50^. All the graphic maps were drawn using MapChart v.2.2 software (Voorrips 2002).

### Phenotypic data and sexual dimorphism

A summary of the descriptive statistics for the metric traits is provided in Table S3. Because sexual dimorphism was bound to affect the morphological variation in our family (*e.g.*, Albert *et al.* 2008; Leinonen *et al.* 2011b), we tested the effect of sex on body shape (for the landmark coordinates) by means of a multivariate analysis of variance. Here, sex, block, and brood were fitted as fixed effects, and Csize as covariate. Separate generalized linear mixed models (GLMMs) for each metric trait also were run with sex and block as fixed factors, Csize as covariate and brood as random effect. GLMM for plate counts was run by using a Poisson instead of normal distribution. For Csize, GLMM was performed with sex and block as fixed factors and brood as a random effect. The analyses were run with the multivariate analysis of variance and LMER procedures using the statistical software R (R Development Core Team 2008). The results revealed that sex had significant effect (*P* < 0.05) on Csize and all the landmark coordinates.

### QTL mapping

A genome-wide scan for QTL was implemented within R/qtl software (Broman *et al.* 2003). The genotypes, phenotypes, and genetic maps (File S1, File S2, and File S3, respectively) converted to four-way cross format were imported with the *read.cross* function. Missing genotypes were first imputed with *fill.geno* function. We used the Haley-Knott regression method (Haley and Knott 1992) to identify QTL. One-dimensional QTL scan (*scanone* function) was initially carried out to detect QTL for landmark coordinates with sex and Csize as covariates. Csize was mapped with sex as a covariate. Although sex did not show significant effect for the metric and meristic traits, it was also included as a covariate for these five traits. Additional QTL were then scanned for each trait using a two-QTL model (*scantwo* function). Finally, a multiple QTL model was fitted to determine QTL for each trait using *makeqtl* and *fitqtl* functions. Locations of QTL were refined with *refineqtl* function. Percentage of phenotypic variance explained (PVE) by each QTL was calculated with *fitqtl* function. The 5% genome-wide significance threshold was estimated by 1000 permutations. QTL were considered to be significant when the LOD scores exceed the 5% genome-wide threshold. Confidence interval (CI) for each QTL peak was derived from the Bayesian 95% credible interval using the *bayesint* function.

## Results

### Segregation analysis

**Figure 2:**
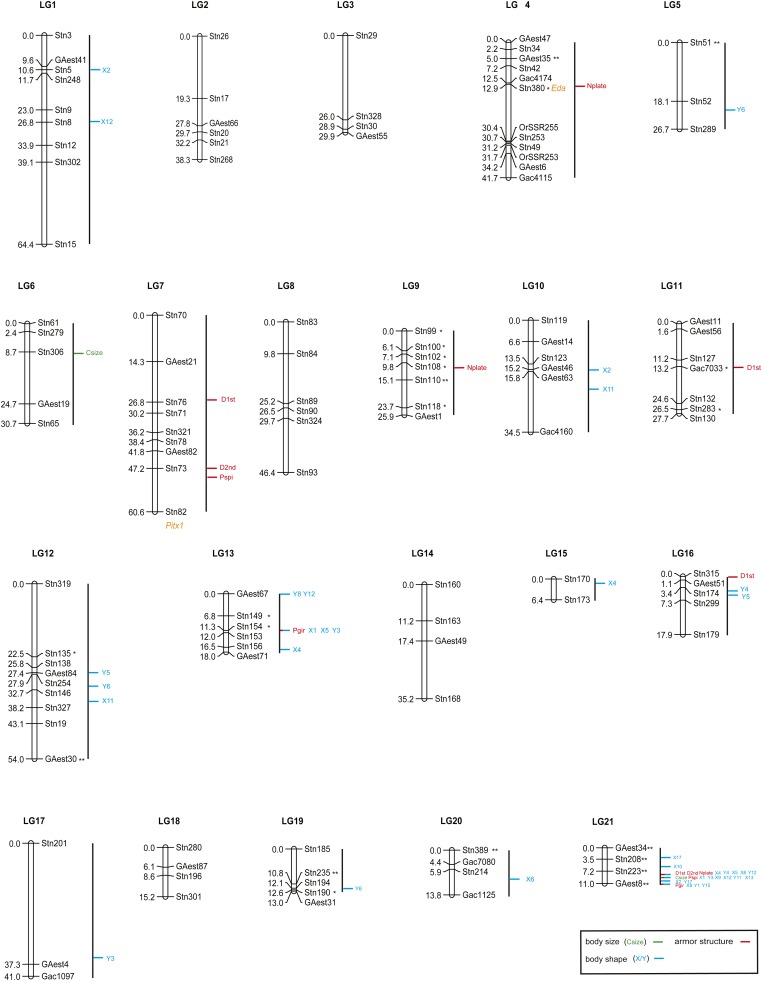
A genome-wide consensus linkage map of *G. aculeatus* and the significant quantitative trait loci detected for phenotypic traits. Traits are divided into body size (centroid size), body shape (landmark coordinates, X/Y), and armor traits with different colors. See Figure 1 for trait abbreviations. The locations of two genes, *Eda* for lateral plate number and *Pitx1* for pelvic development, are shown in orange. *Eda* includes Stn380, and the location of *Pitx1* is below Stn82. Asterisks indicate markers with significantly distorted segregation ratios (*P < 0.05; **P < 0.01).

The F_2_ progeny comprised 190 offspring, 111 of which were females and 79 males. Null alleles were detected in two loci, Stn51 and GAest35. Of the 131 loci, 23 (17.6%) showed significantly distorted segregation ratios (*P* < 0.05; Figure 2). Most of them were scattered across the linkage groups. However, two clusters of distorted loci were found on LG9 (six loci) and LG21 (four loci). Interestingly, most of the distorted loci showed missing alleles in the freshwater grandfather. Despite the potential effect of distorted segregation ratios on genetic linkage map construction and subsequent QTL mapping, all the markers were used in the following analyses because the mapping analysis was implemented in JoinMap using the independence LOD, which is not affected by segregation distortion (Van Ooijen 2006).

### Genetic linkage map

The sex-averaged genetic linkage map is shown in Figure 2. Of the 131 microsatellites, 120 loci were assembled into 21 linkage groups, being consistent with earlier karyotypic (Chen and Reisman 1970; Urton *et al.* 2011) and genetic (*e.g.*, Albert *et al.* 2008; Rogers *et al.* 2012) studies. Of the 11 unmapped loci, six appeared to be unlinked to any other marker with two-point LOD scores ≥ 4.0 and the other five that were assigned to one linkage group were not mapped to any linkage group with LOD scores > 3.0 (see details in Table S2). The orientation of each linkage group was determined by anchoring at least two loci in the published linkage maps of *G. aculeatus* (*e.g.*, Peichel *et al.* 2001; Albert *et al.* 2008). The sizes of the linkage groups ranged from 6.4 cM (LG15) to 64.4 cM (LG1), spanning in total 652.5 cM of the three-spined stickleback genome. The number of markers on each linkage group varied between two (LG15) and 12 (LG4) and the average intermarker interval was 6.6 cM.

### Comparative genomic mapping

A total of 129 loci were assembled onto the 21 chromosomes of *G. aculeatus* with extremely low expectation (E)-values < 1×*e*^-50^ (Figure S1 and Table S2). The physical assignment of loci to chromosomes was consistent with that by genetic linkage analysis with four exceptions (*viz*. Stn34, GAest47, GAest63, and GAest71; Table S2). Comparison of the order of loci on the physical map with that on the genetic linkage map revealed discordance with at least one locus disordered in eight linkage groups/chromosomes (Figure S1).

### QTL mapping

Results of the genetic linkage groups with significant QTL, 95% CI and PVE by QTL for the traits are shown in Figure 2 and Table 1. In total, 52 significant QTL were identified. The number of QTL for each trait ranged from one to four. A dense set of QTL (23) were mapped on LG21, and most of the 95% CIs of QTL overlapped.

**Table 1:** Significant QTL for the meristic, metric traits, and landmark coordinates in three-spine sticklebacks

Trait	LG	Location, cM	Nearest locus, cM	LOD	PVE, %	95% CI, cM
Nplate	4	12.9	Stn380 (12.9)	71.9	74.4	12.9–13.0
	9	12.0	Stn108 (9.8)	18.9	9.1	12.0–12.0
	21	8.0	Stn223 (7.2)	21.3	10.6	7.0–10.0
D1st	7	26	Stn76 (26.8)	8.72	9.05	23.0–26.0
	11	13.2	Gac7033 (13.2)	10.9	11.64	13.0–25.0
	16	0	Stn315 (0.0)	6.0	6.01	0.0–15.0
	21	8	Stn223 (7.2)	34.2	50.55	8.0–9.0
D2nd	7	47.0	Stn73 (47.2)	6.75	7.26	1.0–53.0
	21	8.0	Stn223 (7.2)	36.32	57.64	8.0–10.0
Pspi	7	49.0	Stn73 (47.2)	5.86	6.32	1.0–55.0
	21	9.0	Stn223 (7.2)	35.29	56.16	8.0–10.0
Pgir	13	11.3	Stn154 (11.3)	6.96	10.44	0.0–9.0
	21	11.0	GAest8 (11.0)	19.62	34.60	8.0–11.0
Csize	6	9.0	Stn306 (8.7)	8.92	16.53	5.0–28.0
	21	9.0	Stn223 (7.2)	14.08	27.89	8.0–10.0
X1	13	11.3	Stn154 (11.3)	4.69	9.32	4.0–11.3
	21	9.0	Stn223 (7.2)	8.10	16.81	6.0–11.0
Y1	21	11.0	GAest8 (11.0)	4.84	7.60	6.0–11.0
X2	1	10.6	Stn5 (10.6)	4.88	8.53	5.0–21.0
	10	15.2	GAest46 (15.2)	4.88	8.53	14.0–25.0
	21	10.0	GAest8 (11.0)	10.19	19.06	8.0–11.0
Y3	13	11.3	Stn154 (11.3)	4.61	8.0	6.0–18.0
	17	36.0	GAest4 (37.3)	4.46	7.72	26.0–37.3
	21	9.0	Stn223 (7.2)	4.48	7.78	6.0–11.0
X4	13	17.0	Stn156 (16.5)	6.50	8.92	0.0–18.0
	15	1.0	Stn170 (0.0)	4.36	5.83	0.0–5.0
	20	8.0	Stn214 (5.9)	6.00	8.18	5.9–9.0
	21	8.0	Stn223 (7.2)	16.50	25.82	8.0–10.0
Y4	16	4.0	Stn174 (3.4)	4.58	8.06	0.0–16.0
	21	8.0	Stn223 (7.2)	8.46	15.64	6.0–11.0
X5	13	11.0	Stn154 (11.3)	5.42	7.09	2.0–17.0
	21	8.0	Stn223 (7.2)	24.41	41.05	8.0–9.0
Y5	12	27.4	GAest8 (11.0)	5.82	11.66	25.8–54.0
	16	5.0	Stn174 (3.4)	5.07	10.05	3.0–7.3
X6	21	8.0	Stn223 (7.2)	6.26	12.81	8.0–10.0
Y6	5	20.0	Stn52 (18.1)	5.25	8.85	19.0–25.0
	12	31.0	Stn146 (32.7)	5.38	9.10	27.4–34.0
	19	12.0	Stn194 (12.1)	4.72	7.90	0.0–13.0
X8	21	11.0	GAest8 (11.0)	11.95	24.16	6.0–11.0
Y8	13	0.0	GAest67 (0.0)	4.42	9.89	1.0–35.0
X9	21	9.0	Stn223 (7.2)	8.17	15.07	8.0–11.0
X11	10	20.0	GAest63 (15.8)	5.68	11.52	16.0–33.0
	12	36.0	Stn327 (38.2)	4.67	9.34	35.0–37.0
Y11	21	9.0	Stn223 (7.2)	16.69	32.53	8.0–10.0
X12	1	26.0	Stn8 (26.8)	4.54	7.74	19.0–33.0
	21	9.0	Stn223 (7.2)	12.23	23.51	7.0–11.0
Y12	13	0.0	GAest67 (0.0)	4.79	10.02	0.0–5.0
	21	8.0	Stn223 (7.2)	5.97	12.67	7.2–10.0
X13	21	9.0	Stn223 (7.2)	18.41	34.47	8.0–10.0
Y15	21	11.0	GAest8 (11.0)	6.24	13.07	8.0–11.0
X16	21	11.0	GAest8 (11.0)	5.96	12.70	7.0–11.0
X17	21	3.0	Stn208 (3.5)	5.50	11.89	0.0–9.0

QTL, quantitative trait locus; LG, linkage group; LOD, logarithm of odds; PVE, percentage of phenotypic variance explained; 95% CI, 95% confidence interval. See Figure 1 for trait abbreviations

Two significant QTL were detected for Csize, one QTL on LG21 explaining 27.9% of the variance, and the other on LG6 with PVE value of 16.5%. A total of 13 QTL were detected for the armor traits (Nplate 3, D1st 4, D2nd 2, Pgir 2, and Pspi 2; Table 1). One major QTL with a rather high PVE (34.6–74.4%) and one or several additional QTL with small PVEs (6.0–11.6%) were identified for each of these traits (Table 1). For Nplate, one QTL mapped to locus close to Stn380 on LG4, showed the highest PVE value (74.4%), and the other two QTL had minor effect (9.1–10.6%). Major QTL for the other four armor traits all mapped on LG21 with overlapping 95% CIs, and the PVEs ranged from 34.6 (Pgir) to 57.6% (D2nd).

The X and Y coordinates of each landmark usually mapped to different linkage group regions, implying their distinctiveness in the analyses. A total of 37 significant QTL were found for the 34 X/Y coordinates, 17 of which were mapped to LG21. Of the 37 QTL, 21 were detected for X-coordinates and 16 for Y-coordinates. Ten QTL were found with major effect (PVE > 15%), which were all located on LG21. Eight QTL with major effect were found for X-coordinates.

## Discussion

In the present study, we discovered multiple QTL in the three-spined stickleback, some of which had large phenotypic effects, and many more had moderate-to-small effects. The findings appear to be, to some extent, consistent with those obtained from earlier Pacific crosses of this species, but also many novel QTL were discovered. In what follows, we discuss these issues in more detail and relate our findings to those that have emerged from earlier QTL studies of three-spined sticklebacks.

### QTL mapping of the ecologically important traits

Body size, body shape, and armor structures are ecologically important traits frequently under directional natural and/or sexual selection (Walker 1997; Blanckenhorn 2000; Andersson *et al.* 2006; Head *et al.* 2009). These phenotypic traits show genetically based differentiation among different three-spined stickleback populations (*e.g.*, Wright *et al.* 2004; McGuigan *et al.* 2011; Karhunen *et al.* 2013). During the last decade, studies have started to uncover the genetic bases underlying these traits, especially the armor traits such as the lateral plates and the pelvic structure (Peichel *et al.* 2001; Colosimo *et al.* 2004; Cresko *et al.* 2004; Shapiro *et al.* 2004; Coyle *et al.* 2007). In particular, two major genes—*Ectodysplasin* (*Eda*) and *Pituitary homebox transcription factor* 1 (*Pitx1*)—have been identified to account for plate and pelvic reduction in three-spined sticklebacks, respectively (Shapiro *et al.* 2004; Colosimo *et al.* 2005; Coyle *et al.* 2007; Chan *et al.* 2010).

In this study, 52 significant QTL were identified for traits concerning the body size (centroid size), body shape (X and Y landmark coordinates), and body armor. The pattern of one major plus several minor QTL was observed for most traits, as is the case also in previous studies of sticklebacks (*e.g.*, Colosimo *et al.* 2004; Shapiro *et al.* 2004). One major QTL on LG4 was detected for lateral plate number, with the LOD peak close to Stn380, a marker located within *Eda* gene. This finding is consistent with one earlier study, where one major QTL was also found on LG4 with LOD peak close to marker Gac4174 (Colosimo *et al.* 2004) near the *Eda* gene (see Table S2 for physical locations of Stn380 and Gac4174). However, major QTL for body size, body shape and armor traits (excluding Nplate) were all mapped to LG21, being different from earlier mapping results (Table 2). Such differences also have been observed in previous studies (Table 2). In addition, modifier QTL with smaller effect detected in this and some previous studies also seem to vary between different stickleback populations (Table 2). These differences may be caused by their geographically different parental origins and possibly also by the narrow representation of allelic variation in crosses based on a small number of individuals. Nevertheless, the results also are consistent with the possibility that different genetic architectures may underlie expression of morphological traits in stickleback populations of different geographic origins.

**Table 2:** Comparison of significant QTL for morphological traits used here between this and the previous studies of three-spined sticklebacks

Trait	This Study			Previous Study			
	QTL	LG	PVE, %	Ref*^a^*	QTL	LG	PVE, %
Body size							
Csize/standard body length	Major	LG21	27.9	2	Major	LG19	13
	Minor	LG6	10.4	5		LG13	NA
				6	Major	LG19	20.7
					Minor	LG19	12.1
				7	Major	LG17, LG18	23.6, 41.1
Armor trait							
Nplate	Major	LG4	74.4	1	Major	LG13, LG21*^b^*	26, 23
	Minor	LG9, LG21	9.1−10.6	2	Major	LG4	76.9
					Minor	LG7, LG10, LG21	3.7−6.5
D1st	Major	LG21	50.6	1	Major	LG1, LG2	21, 17
	Minor	LG7, LG11, LG16	6.0−10.9	6	Major	LG9	45.8, 50.4
D2nd	Major	LG21	57.6	1	Major	LG8, LG11	22, 17
	Minor	LG7	7.3				
Pspi	Major	LG21	56.2	1	Major	LG8	25
	Minor	LG7	6.3	3	Major	LG7	65.3, 43.7
					Minor	LG2, LG4	7.6, 5.8
				4	Major	LG7	85.5
Pgir	Major	LG21	34.6	3	Major	LG7	46.8, 27.8
	Minor	LG13	10.4		Minor	LG1, LG2, LG4	5.6−11.1
				4	Major	LG7	87
Body shape							
Coordinate traits	Major	LG21	15.1−41.1	5	Major	LG19, LG7, LG4	15.8−36
	Minor	LG1, LG5, LG10, LG12, LG13, LG5-LG17, LG19-LG21	5.8−13.1		Minor	LG1-5, LG7-LG9, LG12, LG13, LG15-LG21	2.5−14.6
				8	Major	LG4	15.1−40.6
					Minor	LG1, LG2, LG4, LG7-LG14, LG16-LG21	5.8−14.7

Major QTL are defined as PVE > 15%, whereas minor QTL are PVE < 15%. QTL, quantitative trait locus; PVE, percentage of phenotypic variance explained; NA, not available.

a Ref, references including 1: Peichel *et al.* (2001); 2: Colosimo *et al.* (2004); 3: Shapiro *et al.* (2004); 4: Coyle *et al.* (2007); 5: Albert *et al.* (2008); 6: Kitano *et al.* (2009); 7: Greenwood *et al.* (2011); and 8: Rogers *et al.* (2012).

b For comparison, the same linkage group names were used.

As for the QTL effect sizes, their distributions appear to be quite comparable in different stickleback crosses, although the QTL are mapped often to different linkage groups (Table 2; Figure S2). For body size (*i.e.*, centroid size), the major QTL explained ~30% of the variance in this study, and for its other proxy, the standard body length, the major QTL explained 21% and 41% of the variance in two different Pacific crosses (Kitano *et al.* 2009; Greenwood *et al.* 2011). The major QTL for lateral plate number explained more than 70% of the variance in this and one previous study (Colosimo *et al.* 2004). For body shape, several major QTL had PVEs up to 40% in this and two previous studies (Albert *et al.* 2008; Rogers *et al.* 2012). In addition, it seems that the minor QTL for these morphological traits mostly explained ~5–10% of the variance. QTL with PVE below 5% are rare, probably because QTL with small effect size are difficult to detect with genetic mapping approaches such as the one utilized here. Furthermore, it is interesting to observe that the distributions of QTL effect sizes (PVEs) for shape traits between different crosses/studies are similar (Figure S2). This observation supports the conjecture that different chromosomal regions might influence the body shape in different populations, but the general genetic architecture in terms of QTL effect sizes is very similar.

### Multiple QTL on LG21

Among the 21 linkage groups, quite a few QTL (44%) for body size, armor and body shape were mapped on LG21, and many of them appeared to colocalize (*i.e.*, the peak LOD in the same location and the 95% CIs overlapping). It is probably not a coincidence that most of these traits were also significantly intercorrelated at phenotypic level (Table S4). Either pleiotropic effects of a single QTL or tight linkage between genes that influence different morphological traits might contribute to the clustering. Irrespective of the actual cause, the result suggests that the QTL on LG21 may have an important role in controlling many adaptive traits in three-spined sticklebacks. Interestingly, two markers (Stn208, 6.5 Mb; Stn223, 7.5 Mb) on LG21 located close to peaks of these overlapping QTL were in a region where an inversion (5.8–7.5 Mb) on chromosome XXI (*i.e.*, LG21) between Paxton benthic and Japanese Pacific sticklebacks has been recently reported (Jones *et al.* 2012). Several QTL controlling lateral plate numbers (Colosimo *et al.* 2004), body shape traits (Albert *et al.* 2008), and lateral line-related traits (Wark *et al.* 2012) also have been found to map to this same inversion. Given the low marker density on LG21, more markers should be developed and fine-scale mapping should be conducted to better understand the genetic structure of this QTL hotspot.

### QTL estimation bias

Potential biases in the interpretation of QTL effect sizes may be attributable to, for instance, the Beavis effect, referring to overestimation of QTL effects due to small sample sizes (*n* < 500; Beavis 1994, 1998). The Beavis effect is caused by the fact that QTL are reported when the test statistics reach a predetermined threshold, and thus, their estimated effects tend to be upward-biased (Barton and Keightley 2002; Xu 2003). Therefore, increasing the number of individuals and/or markers is expected to detect QTL with smaller effect size (Mackay *et al.* 2009). The Beavis effect can be alleviated by replicating experiments, or by estimating QTL effect sizes from a different sample of individuals instead of that used for QTL detection (Slate 2005).

In three-spined sticklebacks, QTL for similar or identical morphological traits have been identified in some earlier crosses. For instance, comparable distributions of QTL effect size for body shape traits in this and earlier studies (Albert *et al.* 2008; Rogers *et al.* 2012; Table 2 and Figure S2) suggest that the influence of Beavis effect on body shape traits in this study has probably been small. Furthermore, QTL effect sizes for lateral plate number estimated in this study were very similar to those detected in an earlier study (Colosimo *et al.* 2004; Table 2). Therefore, it appears that the Beavis effect might have little influence on the QTL detection power in this study.

### Linkage map *vs.* physical map

Four markers were assigned to linkage groups that differed from the chromosomes in this study. And several conflicts also were detected in marker order between the linkage and physical maps. Several reasons might explain these discrepancies. The first is the accuracy of the linkage mapping approach, which can be affected by factors such as genotyping errors, missing data and/or segregation distortion (Hackett and Broadfoot 2003; Slate 2008). For example, Stn34 was mapped on LG3 in Peichel *et al.* (2001), and on chromosome III in our BLAST searches, but on LG4 in our genetic linkage map. Second, there may be errors in the reference genome sequence (Ross and Peichel 2008; Natri *et al.* 2013; Roesti *et al.* 2013). Third, the disagreements might stem from complex recombination events between divergent genomes, rather than from technical errors. However, some of the loci showing discrepancies (*e.g.*, GAest47, GAest63, and GAest71) were used in the genetic linkage mapping for the first time, and thus, comparisons to other crosses are not possible.

### Distorted segregation ratios of microsatellite markers

The phenomenon that the segregation ratio of a locus deviates from the expected Mendelian ratio is of common occurrence in genome analyses (Lu *et al.* 2002; Xu 2008). Technical problems such as null alleles can yield distorted marker segregation ratios. Here, null alleles were detected in two microsatellite loci (Stn51 and GAest35). As expected, these two loci showed extremely distorted segregation ratios (*P* < 0.01). Alternatively, a variety of genetic and/or physiological factors, including differential transmission in male or female germ line and post-zygotic selection prior to genotypic evaluation (Xu *et al.* 1997), can yield distorted segregation ratios as well. Genetic incompatibility is one of the most common reasons, and increased divergence between species or populations is expected to increase the deviations from the expected Mendelian segregation (Zamir and Tadmor 1986). Although the marine and freshwater *G. aculeatus* populations are phenotypically and genetically divergent, their offspring are highly viable and fertile, as shown in this and earlier studies (*e.g.*, Peichel *et al.* 2001; Colosimo *et al.* 2004; Shapiro *et al.* 2004; Albert *et al.* 2008; Rogers *et al.* 2012). Therefore, genetic incompatibilities seem to be an unlikely explanation in our case.

Presence of segregation distortion loci (SDL; Vogl and Xu 2000) also could lead to distorted segregation ratios of molecular markers. SDL are loci that are subject to gametic or zygotic selection and consequently cause segregation distortion (Xu 2008). Loci linked to SDL can be indirectly distorted because of genetic hitchhiking (Xu *et al.* 1997), thus leading to clustering of distorted loci. This phenomenon is detected on LG9 and LG21 in our linkage map. The clustering of the distorted markers suggests that deleterious recessive alleles are probably linked to them (Zamir and Tadmor 1986). Regions with three or more closely linked loci that exhibit significant segregation distortion are termed segregation distortion regions (SDRs), and identifying common SDRs among different populations would be helpful to determine the underlying SDL (Lu *et al.* 2002). However, we are not certain that the two regions are actually common SDRs among different three-spined stickleback crosses, because no distorted markers have ever been reported in the genetic linkage maps of the three-spined stickleback (*e.g.*, Peichel *et al.* 2001; Albert *et al.* 2008; Rogers *et al.* 2012).

In summary, we identified multiple QTL for a set of ecologically important morphological traits that are known to exhibit significant differentiation between marine and freshwater stickleback populations. The results identified several significant QTL with large effects in several morphological traits, as well as a large number of QTL with smaller effects. In accordance with earlier studies, a QTL on LG4 was identified to have a major effect on the lateral plate number. In addition, several major overlapping QTL were mapped on LG21, suggesting pleiotropic effects or tight genetic linkage between QTL. These QTL were associated with centroid size, lengths of pelvic girdle, pelvic and dorsal spines, and different aspects of body shape, implying the potential role of genomic segments on LG21 in multiple adaptive processes. Further studies are needed to identify candidate genes on this linkage group (*i.e.*, chromosome 21) controlling the correlated complex morphological traits of three-spined stickleback. All in all, this study provides new insights into genetic architecture of adaptive morphological traits in three-spined sticklebacks and should aid further studies aimed at deciphering the genetic basis of morphological variation in this species.

## Supplementary Material

Supporting Information
