# Supplementary material for: Identification of Major and Minor QTL for Ecologically Important Morphological Traits in Three-Spined Sticklebacks (Gasterosteus aculeatus)
Source: G3 (Bethesda). 2014 Feb 13;4(4):595–604. doi: 10.1534/g3.114.010389 (PMC4059232; doi:10.1534/g3.114.010389)
Supplement: Supporting Information [file supp_4_4_595__index.html]

Identification of Major and Minor QTL for Ecologically Important Morphological Traits in Three-Spined Sticklebacks (Gasterosteus aculeatus) — Supporting Information 

# Identification of Major and Minor QTL for Ecologically Important Morphological Traits in Three-Spined Sticklebacks (*Gasterosteus aculeatus*)

## Supporting Information for Liu *et al.*, 2014

**Files in this Data Supplement:**

- Supporting Information - Figures S1-S2, Tables S1-S4, and Files S1-S3 (PDF, 199 KB)
- Figure S1 - Comparison of microsatellite loci on genetic linkage map (black) versus physical map (red). (PDF, 474 KB)
- Figure S2 - The distribution of the QTL effect sizes as the percentage of phenotypic variance explained (PVE) in A) this study; B) Albert *et al.* (2008); C) Rogers *et al.* (2012). (PDF, 65 KB)
- Table S1 - Summary of QTL mapping results from earlier studies of the three-spined stickleback, including morphological traits investigated, crossing design (number of the individuals used for mapping), origins of the parents, marker type and number, size of the linkage map constructed, number of QTL detected, and the maximum and average values of the percentage of variance explained by the QTL (PVE). (PDF, 71 KB)
- Table S2 - Summary of BLAST search results of the microsatellite loci. (PDF, 122 KB)
- Table S3 - Summary statistics of the meristic and metric traits. (PDF, 112 KB)
- Table S4 - Spearman rank correlations among traits in F2 progeny. For trait abbreviations, see Figure 1. (.xls, 53 KB)
- File S1 - Raw genotyping data (.txt, 196 KB)
- File S2 - Raw phenotypic data (.txt, 85 KB)
- File S3 - Raw map data (.txt, 1 KB)
