# Supplementary material for: Identification of Major and Minor QTL for Ecologically Important Morphological Traits in Three-Spined Sticklebacks (Gasterosteus aculeatus)
Source: G3 (Bethesda). 2014 Feb 13;4(4):595–604. doi: 10.1534/g3.114.010389 (PMC4059232; doi:10.1534/g3.114.010389)
Supplement: Supporting Information [file supp_g3.114.010389_010389SI.pdf]

G3·Genes | Genomes | Genetics

Identification of major and minor QTL for ecologically important morphological traits in three-spined sticklebacks (*Gasterosteus aculeatus)*

Jun Liu*, Takahito Shikano†, Tuomas Leinonen†, José Manuel Cano‡, Meng-Hua Li*and Juha Merilä †

*CAS Key Laboratory of Animal Ecology and Conservation Biology, Institute of Zoology, Chinese Academy of Sciences (CAS), Beijing 100101, China

†Ecological Genetics Research Unit, Department of Biosciences, P.O. Box 65, FIN-00014 University of Helsinki, Finland

‡Research Unit of Biodiversity (UO-CSIC-PA), Edificio de Investigación, C/Gonzalo Gutiérrez Quirós s/n, 33600, Mieres, Spain

Corresponding author: Meng-Hua Li, CAS Key Laboratory of Animal Ecology and Conservation Biology, Institute of Zoology, Chinese Academy of Sciences (CAS), Beichen West Road No. 1-5, Chaoyang District, Beijing 100101, China. Tel: +86-10-64806336; Fax: +86-10-64806336; E-mail: menghua.li@ioz.ac.cn

DOI: 10.1534/g3.114.010389

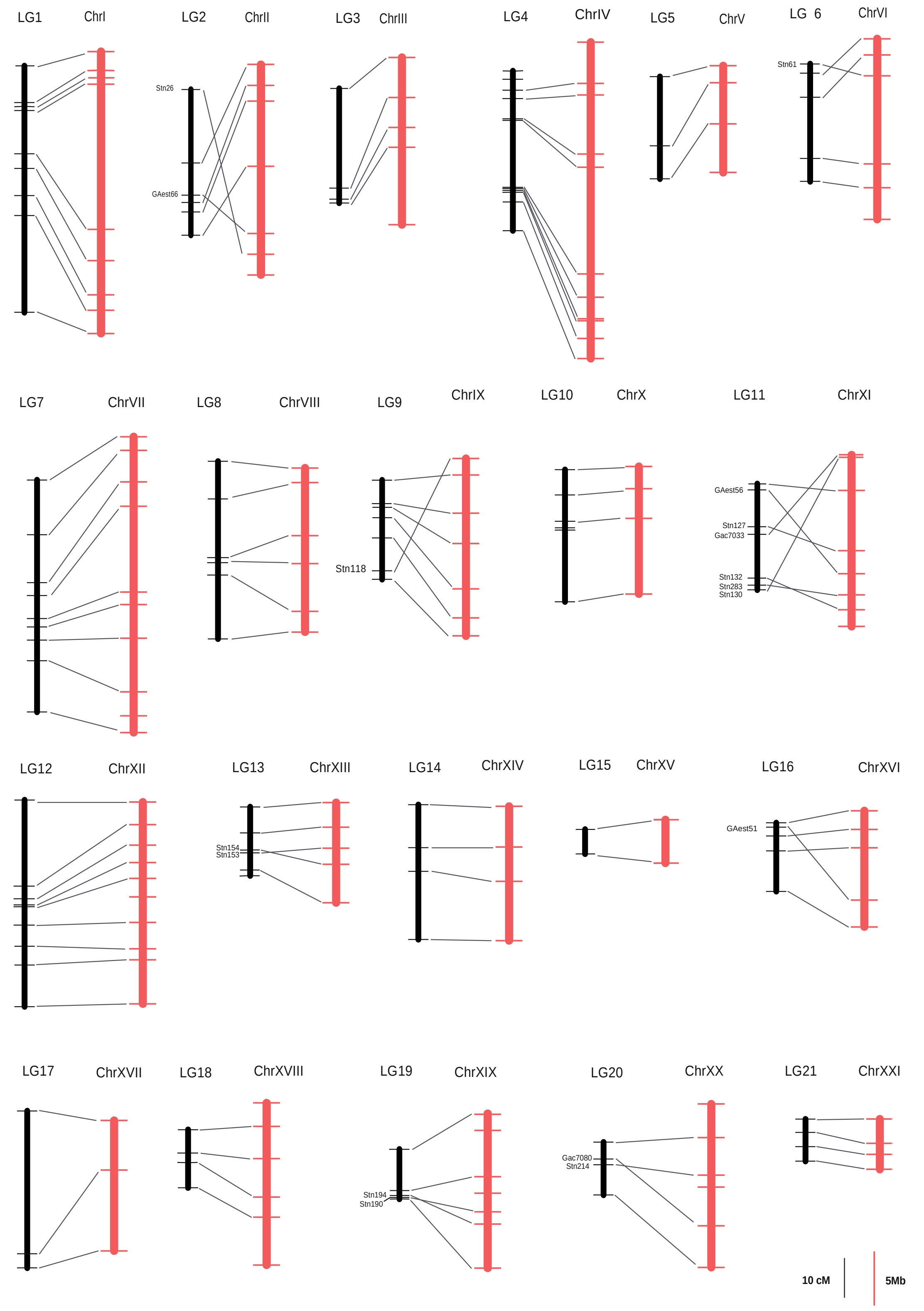

Figure S1 Comparison of microsatellite loci on genetic linkage map (black) versus physical map (red). The markers on the same linkage/chromosome that do not match between the two maps are shown. The physical map is drawn according to the BLAST searches against the *G. aculeatus* genome assembly (Roesti *et al.* 2013).

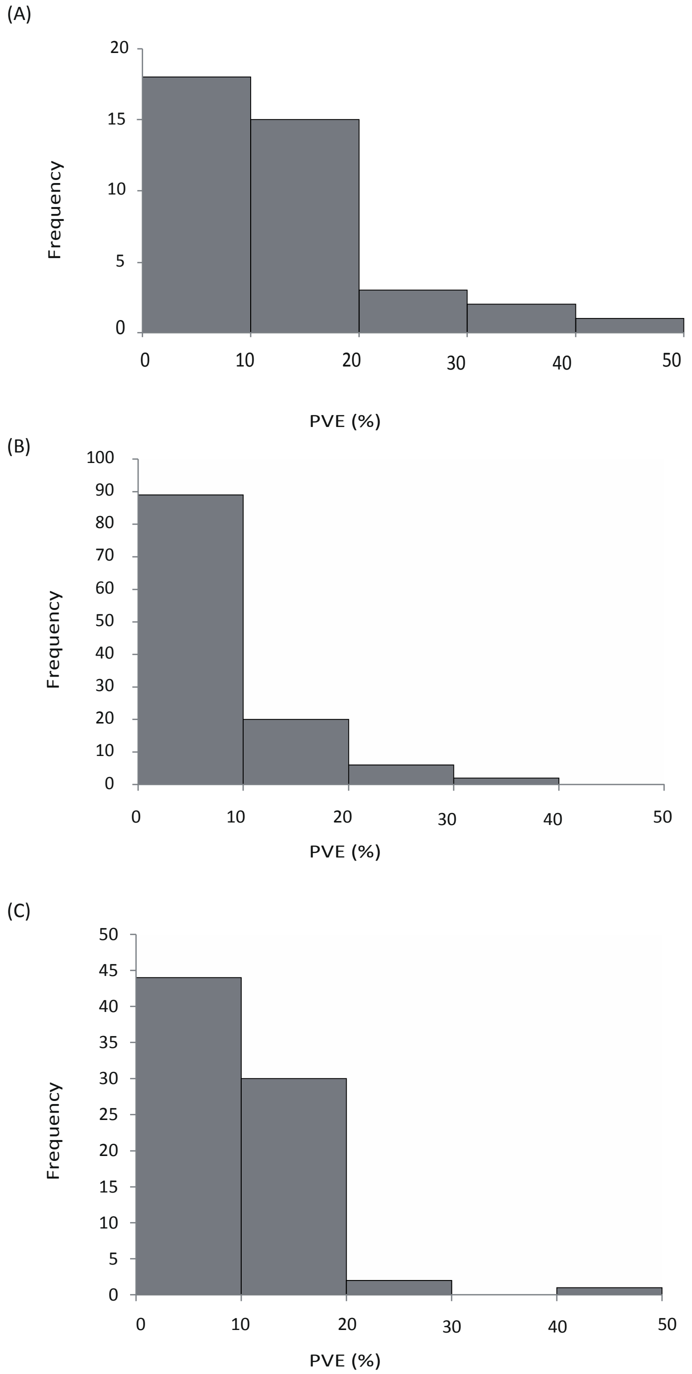

Figure S2 The distribution of the QTL effect sizes as the percentage of phenotypic variance explained (PVE) in A) this study; B) Albert *et al.* (2008); C) Rogers *et al.* (2012).

Table S1 Summary of QTL mapping results from earlier studies of the three-spined stickleback, including morphological traits investigated, crossing design (number of the individuals used for mapping), origins of the parents, marker type and number, size of the linkage map constructed, number of QTL detected, and the maximum and average values of the percentage of variance explained by the QTL (PVE).

Traits	Cross (No.)	Grandmother	Grandfather	Marker		Map distance	QTL	PVE (%)		References
				type	No.	(cM)	No.	Max	Ave	
Gill raker number, lateral plate number, dorsal spine 1 and 2 lengths, and pelvic spine length	F_2 (92)	Benthic female, Priest lake, British Columbia	Limnetic male, Priest lake, British Columbia	SSR	227	886	9	37	24.5	Peichel *et al.* 2001
Pattern, number, and size of bony lateral plates	F_2 (360)	Marine female, Japan	Benthic male, Paxton Lake, British Columbia.	SSR	160	NA	20	77.6	19.3	Colosimo *et al.* 2004
Lateral plate morph, and pelvic morph	F_2 (98)	Bear Paw lake, Alaska	Rabbit Slough, Alaska	SSR	NA	NA	2	NA	NA	Cresko *et al.* 2004
Pelvic morph, pelvic spine length, pelvic girdle length, ascending branch height, asymmetry	F_2 (375)	Marine female, Japan	Benthic, Paxton Lake, British Columbia	SSR	NA	NA	16	65.3	24.1	Shapiro *et al.* 2004
Area (As) and two length components (VPs and JPs) of opercle	F_2 (99)	Anadromous fish from Rabbit Slough, Alaska	Bear Paw lake, Alaska	SSR	NA	NA	2	> 30	NA	Kimmel *et al*. 2005

Pelvic spine number, pelvic spine length, pelvic girdle length, asymmetry	F_2 (177)	Loch Fada, North Uist, Scotland	River Kelvin, Glasgow, Scotland	SSR	6	NA	5	96.6	78.5	Coyle *et al.* 2007
Gill pigmentation	F_2 (360)	Marine female, Japan	Benthic, Paxton Lake, British Columbia.	SSR	NA	NA	1	56.1	NA	Miller *et al.* 2007
Body shape: 54 landmark coordinates	F_2 (372)	Marine female, Japan	Paxton Lake, Texada Island, British Columbia	SSR	248	1220	117	30.2	9	Albert *et al*. 2008
Sperm number, testis size, body length and weight, mean and max dorsal pricking and first dorsal spine length	backcross (76 males)	Japan Sea female	Pacific Ocean male	SSR and SNP	14 SSR; 90 SNP	NA	8	52.6	33.3	Kitano *et al.* 2009
	F_2 (70 males)	Pacific Ocean female	Japan Sea male	SSR and SNP	9 SSR; 138 SNP	NA	7	50.4	20.9	
Degree of barring, midline light bar melanophores, dorsal and dorsal melanophores and standard length	F_2 (176)	Hotel Lake, British Columbia	Anadromous marine, Little Campbell River, British Columbia	SSR and SNP	1 SSR; 279 SNP	1217	9	41.1	15.2	Greenwood *et al.* 2011
Body shape: 54 landmark coordinates	Four F_2 populations (374; 374; 290; 361)	Marine population in southwestern British Columbia,	Freshwater lakes in southwestern British Columbia,	SSR	250 (96; 94; 75; 85)	993 (average)	77	40.6	10.8	Rogers *et al*. 2012

Sensory neuromast number, neuromast pattern, groove morphology, the presence of lateral plates and pelvic structures:16 traits in total	F_2 (234)	Benthic female, Paxton Lake ,British Columbia	Marine male, Japan	SNP	245	1061	23	80	26.7	Wark *et al.* 2012

NA, not available.

SSR, microsatellite.

SNP, single nucleotide polymorphism.

Table S2 Summary of BLAST search results of the microsatellite loci. GenBank accession number (No.), genomic location (start base pair position [bp] on chromosome), E-value, assignment on genetic linkage map (LG) and location (cM) on the LG are shown. Markers in bold are those assigned to different chromosomes from the corresponding linkage groups. Note that since two hits (both E-values = 0) are detected for Stn290, this locus is not used in the physical map.

Marker	GenBank No.	Chromosome	Start location (bp)	E-value	LG	location (cM)
Stn3	G72128	I	2434762	0	1	0
GAest41	DN666064	I	4177759	0	1	9.6
Stn5	G72328	I	4860953	0	1	10.6
Stn248	BV678074	I	5455664	0	1	11.7
Stn9	G72131	I	18878483	0	1	23
Stn8	G72130	I	21776796	0	1	26.8
Stn12	G72132	I	24932820	0	1	33.9
Stn302	BV678112	I	26362882	0	1	39.1
Stn15	G72236	I	28513520	1.0E-104	1	64.4
Stn17	G72237	II	3673872	7.0E-66	2	19.3
Stn20	G72238	II	5614532	0	2	29.7
Stn21	G72136	II	7049414	0	2	32.2
Stn268	BV678088	II	13092167	0	2	38.3
GAest66	DN683768	II	19316163	1.0E-108	2	27.8
Stn26	G72240	II	21231787	1.0E-140	2	0
GAest36†	DN687963	II	23153000	0	2	
Stn29	G72140	III	912868	1.0E-135	3	0
Stn328	BV678131	III	4621956	0	3	26
Stn30	G72241	III	7388905	1.0E-166	3	28.9
GAest55	DN698980	III	9238989	0	3	29.9
**Stn34**	G72243	III	16391206	1.0E-160	4	2.2
Stn37	G72144	IV	1228632	0	-	
GAest35	DN685500	IV	5043077	0	4	5
Stn42	G72148	IV	6107399	0	4	7.2
Gac4174	AJ010358	IV	11585867	1.0E-90	4	12.5
Stn380‡		IV	12800220		4	12.9
OrSSR255	BV686585	IV	22671119	1.0E-103	4	30.4
Stn253	BV678078	IV	24822934	0	4	30.7
Stn49	G72153	IV	26827104	4.0E-96	4	31.2
OrSSR253	BV686584	IV	26998500	1.0E-173	4	31.7
GAest6	DN733971	IV	28629504	0	4	34.2
Gac4115	AJ311858	IV	30495507	0	4	41.7
Stn51	G72248	V	4860953	0	5	0
Stn52	G72154	V	6426588	0	5	18.1
Stn289	BV678100	V	10240359	2.0E-79	5	26.7

Stn59	G72156	V	14745396	0	-	
Stn279	BV678153	VI	1079376	1.0E-79	6	2.4
Stn306	BV678158	VI	2576468	1.0E-137	6	8.7
Stn61	G72158	VI	4512139	5.0E-86	6	0
GAest19	DN728221	VI	12657122	0	6	24.7
Stn65	G72254	VI	14859529	0	6	30.7
Stn67†	G72161	VI	17788389	1.0E-128	6	
Stn70	G72164	VII	1038649	0	7	0
GAest21	DN704336	VII	2300505	0	7	14.3
Stn76	G72259	VII	5222127	0	7	26.8
Stn71	G72256	VII	7478387	1.0E-105	7	30.2
Stn321	BV678125	VII	15412060	0	7	36.2
Stn78	G72261	VII	16567658	0	7	38.4
GAest82	DN706699	VII	19686426	0	7	41.8
Stn73	G72326	VII	24649300	0	7	47.2
Stn257	BV678148	VII	26855892	0	-	
Stn82	G72168	VII	28409267	0	7	60.6
Stn83	G72263	VIII	727015		8	0
Stn84	G72169	VIII	2070758	0	8	9.8
Stn89	G72265	VIII	6987124		8	25.2
Stn90	G72173	VIII	9574730	1.0E-95	8	26.5
Stn324	BV678164	VIII	13983521	1.0E-134	8	29.7
Stn93	G72266	VIII	15899964	0	8	46.4
Stn118	G72186	IX	611015	0	9	23.7
Stn99	G72270	IX	2131460	0	9	0
Stn100	G72177	IX	5677783	0	9	6.1
Stn102	G72179	IX	8476129	0	9	7.1
Stn108	G72181	IX	12668166	0	9	9.8
Stn110	G72182	IX	15365445	0	9	15.1
GAest1	DN712245	IX	17020716	0	9	25.9
Stn119	G72280	X	2351806	1.0E-144	10	0
GAest14	DN735932	X	4408367	0	10	6.6
Stn123	G72188	X	7152473	1.0E-115	10	13.5
GAest46	Na				10	15.2
Gac4160	AJ311859	X	14148435	0	10	34.5
Gac7033	AJ010360	XI	219488	0	11	13.2
Stn130	G72286	XI	456012	1.0E-120	11	27.7
GAest11	DN735398	XI	3514008	1.0E-169	11	0
Stn127	G72191	XI	9085803	0	11	11.2

GAest56	DN683451	XI	11210419	0	11	1.6
Stn283	BV678099	XI	13179760	0	11	26.5
Stn132	G72193	XI	14561862	0	11	24.6
GAest80†	DN704459	XI	16106586	8.0E-99	11	
Stn319	BV678123	XII	263868	0	12	0
Stn135	G72288	XII	2354213	0	12	22.5
Stn138	G72291	XII	4247384	0	12	25.8
GAest84	DN732607	XII	5850508	0	12	27.4
Stn254	BV678079	XII	7327889	1.0E-134	12	27.9
**GAest71**	DN713857	XII	9027112	0	13	18
Stn146	G72296	XII	11409165	0	12	32.7
Stn327	BV678130	XII	13826118	0	12	38.2
Stn19	G72135	XII	14859628	0	12	43.1
GAest30	DN685788	XII	18935717	0	12	54
GAest67	DN685529	XIII	3913576	0	13	0
Stn149	G72199	XIII	6175858	0	13	6.8
Stn153	G72202	XIII	8142821	0	13	12
Stn154	G72203	XIII	9617733	0	13	11.3
Stn156	G72204	XIII	13180379	0	13	16.5
Stn160	G72301	XIV	2283168	0	14	0
Stn163	G72304	XIV	6057628	0	14	11.2
GAest49	DN730395	XIV	9232964	0	14	17.4
Stn168	G72209	XIV	14713222	0	14	35.2
Stn170	G72307	XV	6134163	0	15	0
Stn173	G72309	XV	10145445	0	15	6.4
Stn315	BV678120	XVI	4544023	0	16	0
Stn174	G72310	XVI	6274460	0	16	3.4
Stn299	BV678109	XVI	7973363	0	16	7.3
GAest51	DN728002	XVI	12808591	0	16	1.1
Stn179	G72212	XVI	15284747	1.0E-111	16	17.9
Stn201	G72225	XVII	873872	1.0E-123	17	0
GAest4	DN736839	XVII	5471871	0	17	37.3
Gac1097	AJ010352	XVII	12950114	1.0E-149	17	41
Gac7148†	AJ311865	XVIII	681853	0	18	
Stn280	BV678096	XVIII	2847443	1.0E-126	18	0
GAest87	DN687948	XVIII	5850220	0	18	6.1
Stn196	G72320	XVIII	9393373	0	18	8.6
Stn301	BV678111	XVIII	11250093	0	18	15.2

Stn308	BV678159	XVIII	15688564	1.0E-145	-	
Stn290	BV678101	XIX	19858	0	-	
		ChrUn	29507624	0		
Stn185	G72214	XIX	1643763	0	19	0
**GAest47**	DN705394	XIX	3121741	0	4	0
Stn235	BV678166	XIX	7397482	0	19	10.8
Stn263†	BV678150	XIX	8937897	0	19	
Stn190	G72217	XIX	10661888	1.0E-100	19	12.6
Stn194	G72220	XIX	11789382	0	19	12.1
GAest31	DN685475	XIX	15863729	0	19	13
Stn204	G72323	XX	891836	1.0E-177	-	
Stn389	BV678142	XX	4004182	1.0E-180	20	0
Stn214	BV102492	XX	7483155	1.0E-176	20	5.9
**GAest63**	DN693003	XX	8579616	0	10	
Gac7080	AJ311864	XX	12173103	0	20	4.4
Gac1125	AJ010354	XX	16009419	0	20	13.8
GAest34	DN682722	XXI	4278648	0	21	0
Stn208	G72229	XXI	6525514	0	21	3.5
Stn223	BV102499	XXI	7545763	1.0E-162	21	7.2
GAest8	DN732699	XXI	8938069	0	21	11

-, marker that was not assigned to any linkage group.

†, marker that was assigned to one linkage group, but not mapped on the linkage map.

‡, the location of Stn380 on chromosome was determined by the location of *Eda* gene (http://asia.ensembl.org/).

Na, no sequence information available.

Table S3 Summary statistics of the meristic and metric traits. For trait abbreviations, see Figure. 1.

Trait	No.	Mean	SD	min	max	CV (%)
Nplate	190	30	13	4	65	46.4
D1st (mm)	181	2.71	0.442	1.14	4.20	16.3
D2nd (mm)	190	2.94	0.478	1.25	4.34	16.2
Pspi (mm)	189	3.80	0.700	1.22	5.50	18.4
Pgir (mm)	190	5.49	0.729	2.76	7.38	13.3
Csize	185	3.76	0.37	2.78	4.88	9.8

No., number of individuals measured for each trait.

SD, standard deviation.

min, minimum value measured for one trait.

max, maximum value measured for one trait.

CV, coefficient of variation.

Table S4 Spearman rank correlations among traits in F_2 progeny. For trait abbreviations, see Figure 1.

Available for download as an Excel file at http://www.g3journal.org/lookup/suppl/doi:10.1534/g3.114.010389/-/DC1

Files S1-S3

Available for download as .txt files at http://www.g3journal.org/lookup/suppl/doi:10.1534/g3.114.010389/-/DC1

File S1 Raw genotyping data.

File S2 Raw phenotypic data.

File S3 Raw map data.
