# Supplementary material for: Identification of Major and Minor QTL for Ecologically Important Morphological Traits in Three-Spined Sticklebacks (Gasterosteus aculeatus)
Source: G3 (Bethesda). 2014 Feb 13;4(4):595–604. doi: 10.1534/g3.114.010389 (PMC4059232; doi:10.1534/g3.114.010389)
Supplement: Supporting Information [file supp_g3.114.010389_FigureS1.pdf]

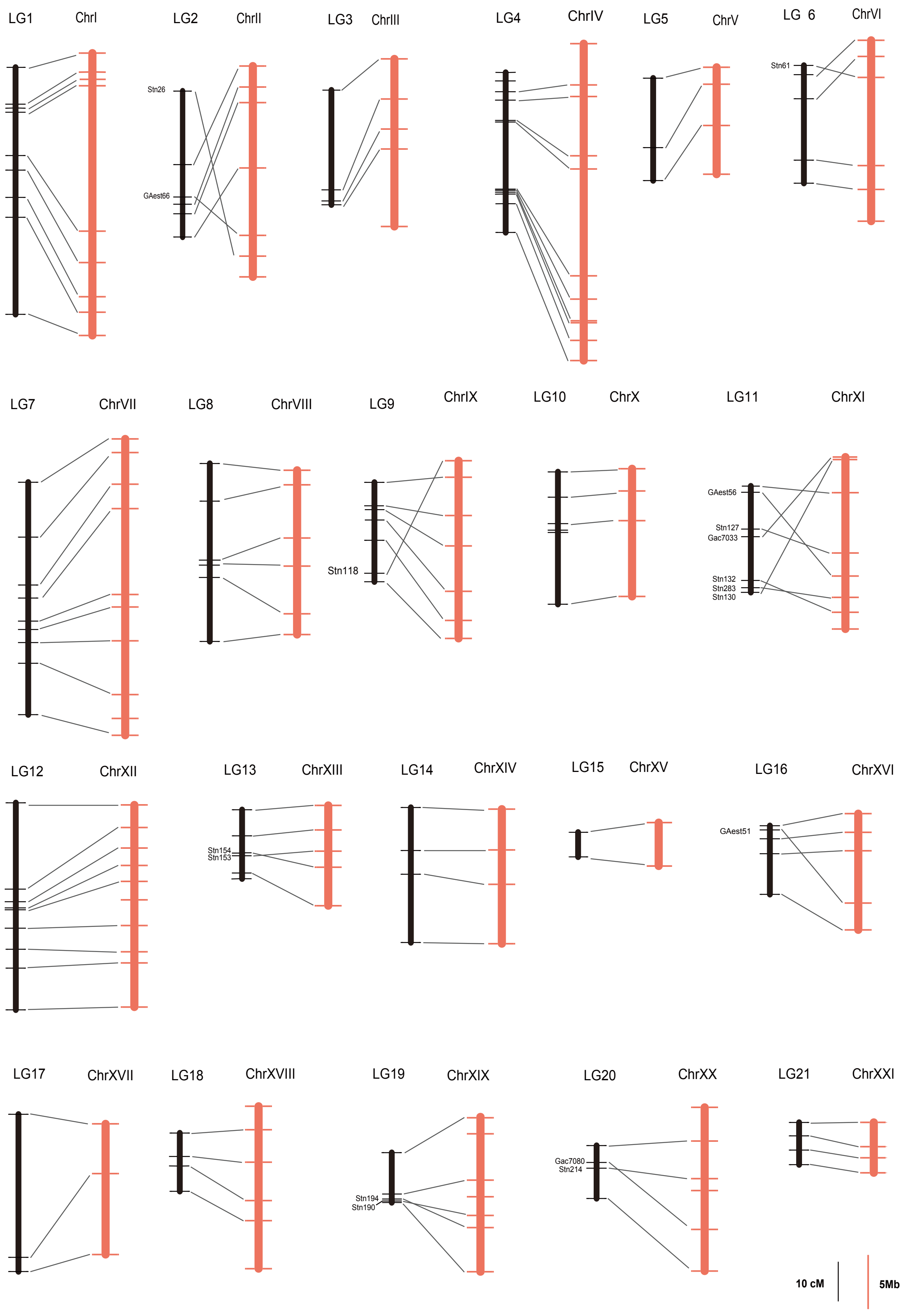

Figure S1 Comparison of microsatellite loci on genetic linkage map (black) versus physical map (red). The markers on the same linkage/chromosome that do not match between the two maps are shown. The physical map is drawn according to the BLAST searches against the *G. aculeatus* genome assembly (Roesti *et al.* 2013).
