# Supplementary material for: Identification of Major and Minor QTL for Ecologically Important Morphological Traits in Three-Spined Sticklebacks (Gasterosteus aculeatus)
Source: G3 (Bethesda). 2014 Feb 13;4(4):595–604. doi: 10.1534/g3.114.010389 (PMC4059232; doi:10.1534/g3.114.010389)
Supplement: Supporting Information [file supp_g3.114.010389_FigureS2.pdf]

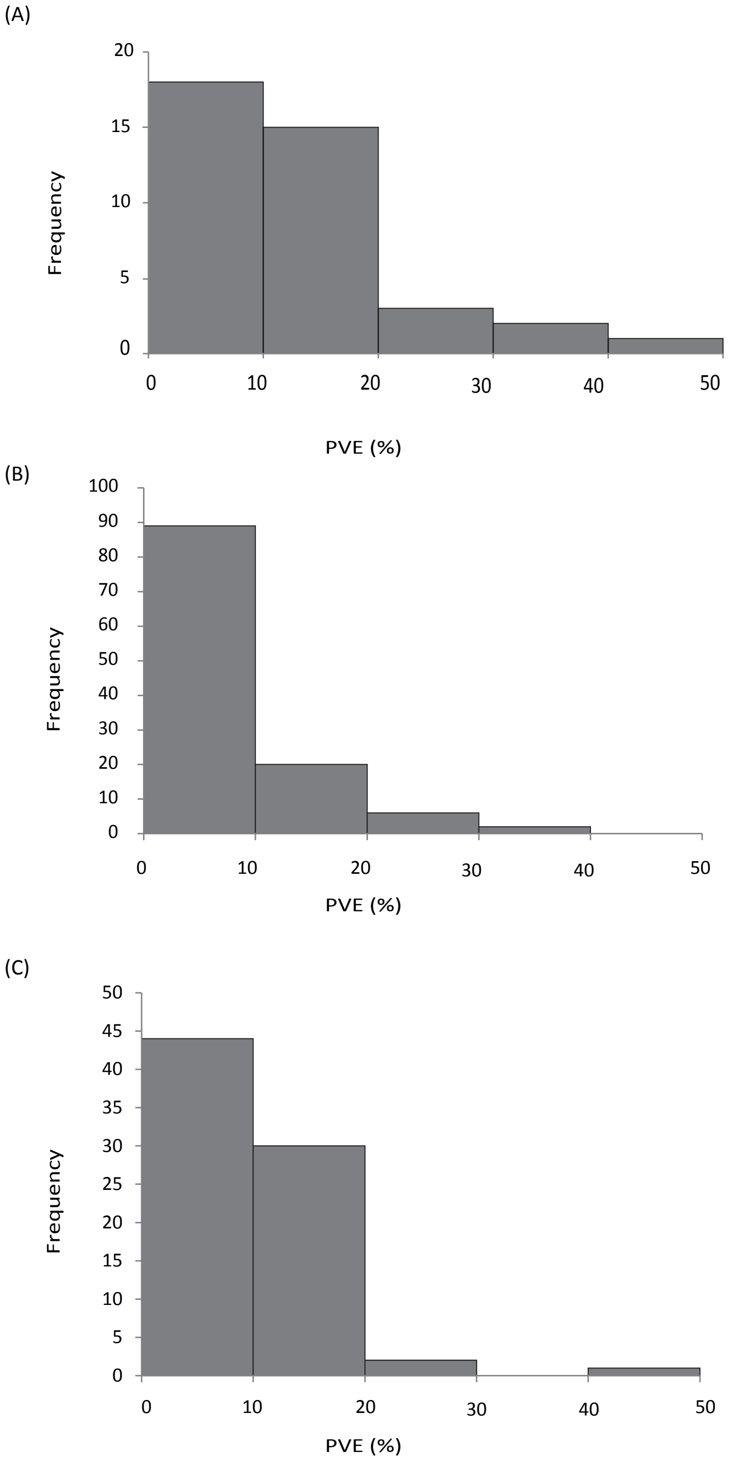

Figure S2 The distribution of the QTL effect sizes as the percentage of phenotypic variance explained (PVE) in A) this study; B) Albert *et al.* (2008); C) Rogers *et al.* (2012).
