# Supplementary material for: Identification of Major and Minor QTL for Ecologically Important Morphological Traits in Three-Spined Sticklebacks (Gasterosteus aculeatus)
Source: G3 (Bethesda). 2014 Feb 13;4(4):595–604. doi: 10.1534/g3.114.010389 (PMC4059232; doi:10.1534/g3.114.010389)
Supplement: Supporting Information [file supp_g3.114.010389_TableS1.pdf]

Table S1 Summary of QTL mapping results from earlier studies of the three-spined stickleback, including morphological traits investigated, crossing design (number of the individuals used for mapping), origins of the parents, marker type and number, size of the linkage map constructed, number of QTL detected, and the maximum and average values of the percentage of variance explained by the QTL (PVE).

Traits	Cross (No.)	Grandmother	Grandfather	Marker		Map distance	QTL	PVE (%)		References
				type	No.	(cM)	No.	Max	Ave	
Gill raker number, lateral plate number, dorsal spine 1 and 2 lengths, and pelvic spine length	F_2 (92)	Benthic female, Priest lake, British Columbia	Limnetic male, Priest lake, British Columbia	SSR	227	886	9	37	24.5	Peichel *et al.* 2001
Pattern, number, and size of bony lateral plates	F_2 (360)	Marine female, Japan	Benthic male, Paxton Lake, British Columbia.	SSR	160	NA	20	77.6	19.3	Colosimo *et al.* 2004
Lateral plate morph, and pelvic morph	F_2 (98)	Bear Paw lake, Alaska	Rabbit Slough, Alaska	SSR	NA	NA	2	NA	NA	Cresko *et al.* 2004
Pelvic morph, pelvic spine length, pelvic girdle length, ascending branch height, asymmetry	F_2 (375)	Marine female, Japan	Benthic, Paxton Lake, British Columbia	SSR	NA	NA	16	65.3	24.1	Shapiro *et al.* 2004
Area (As) and two length components (VPs and JPs) of opercle	F_2 (99)	Anadromous fish from Rabbit Slough, Alaska	Bear Paw lake, Alaska	SSR	NA	NA	2	> 30	NA	Kimmel *et al*. 2005

Pelvic spine number, pelvic spine length, pelvic girdle length, asymmetry	F_2 (177)	Loch Fada, North Uist, Scotland	River Kelvin, Glasgow, Scotland	SSR	6	NA	5	96.6	78.5	Coyle *et al.* 2007
Gill pigmentation	F_2 (360)	Marine female, Japan	Benthic, Paxton Lake, British Columbia.	SSR	NA	NA	1	56.1	NA	Miller *et al.* 2007
Body shape: 54 landmark coordinates	F_2 (372)	Marine female, Japan	Paxton Lake, Texada Island, British Columbia	SSR	248	1220	117	30.2	9	Albert *et al*. 2008
Sperm number, testis size, body length and weight, mean and max dorsal pricking and first dorsal spine length	backcross (76 males)	Japan Sea female	Pacific Ocean male	SSR and SNP	14 SSR; 90 SNP	NA	8	52.6	33.3	Kitano *et al.* 2009
	F_2 (70 males)	Pacific Ocean female	Japan Sea male	SSR and SNP	9 SSR; 138 SNP	NA	7	50.4	20.9	
Degree of barring, midline light bar melanophores, dorsal and dorsal melanophores and standard length	F_2 (176)	Hotel Lake, British Columbia	Anadromous marine, Little Campbell River, British Columbia	SSR and SNP	1 SSR; 279 SNP	1217	9	41.1	15.2	Greenwood *et al.* 2011
Body shape: 54 landmark coordinates	Four F_2 populations (374; 374; 290; 361)	Marine population in southwestern British Columbia,	Freshwater lakes in southwestern British Columbia,	SSR	250 (96; 94; 75; 85)	993 (average)	77	40.6	10.8	Rogers *et al*. 2012

Sensory neuromast number, neuromast pattern, groove morphology, the presence of lateral plates and pelvic structures:16 traits in total	F_2 (234)	Benthic female, Paxton Lake ,British Columbia	Marine male, Japan	SNP	245	1061	23	80	26.7	Wark *et al.* 2012

NA, not available.

SSR, microsatellite.

SNP, single nucleotide polymorphism.
