# Supplementary material for: Identification of Major and Minor QTL for Ecologically Important Morphological Traits in Three-Spined Sticklebacks (Gasterosteus aculeatus)
Source: G3 (Bethesda). 2014 Feb 13;4(4):595–604. doi: 10.1534/g3.114.010389 (PMC4059232; doi:10.1534/g3.114.010389)
Supplement: Supporting Information [file supp_g3.114.010389_TableS3.pdf]

Table S3 Summary statistics of the meristic and metric traits. For trait abbreviations, see Figure. 1.

Trait	No.	Mean	SD	min	max	CV (%)
Nplate	190	30	13	4	65	46.4
D1st (mm)	181	2.71	0.442	1.14	4.20	16.3
D2nd (mm)	190	2.94	0.478	1.25	4.34	16.2
Pspi (mm)	189	3.80	0.700	1.22	5.50	18.4
Pgir (mm)	190	5.49	0.729	2.76	7.38	13.3
Csize	185	3.76	0.37	2.78	4.88	9.8

No., number of individuals measured for each trait.

SD, standard deviation.

min, minimum value measured for one trait.

max, maximum value measured for one trait.

CV, coefficient of variation.
